# ‘The medical gaze’: Foucault, anthropology and contemporary psychiatry in Ireland

**DOI:** 10.1007/s11845-021-02725-w

**Published:** 2021-08-10

**Authors:** Aoife K O’Callaghan

**Affiliations:** Department of Psychiatry, Trinity Centre for Health Sciences, Tallaght University Hospital, Dublin 24, Dublin, D24 NR0A Ireland

**Keywords:** Foucault, Medical anthropology, Psychiatry

## Abstract

Michel Foucault developed the concept of ‘the medical gaze’, describing how doctors fit a patient’s story into a ‘biomedical paradigm, filtering out what is deemed as irrelevant material’ (Misselbrook, 2013). Doctors are perceived within this model to focus on selecting the biomedical elements of patients’ problems only, filtering out all other elements of a person’s life story, but this paper argues that in the subspecialty of psychiatry, this is not the case, and such a filter is not so easily applied.

## Introduction

What is the medical gaze? Michel Foucault developed the concept of ‘the medical gaze’, describing how doctors fit a patient’s story into a ‘biomedical paradigm, filtering out what is deemed as irrelevant material’ [1]. Doctors are perceived within this model to focus on selecting the biomedical elements of patients’ problems, but this paper argues that in the subspecialty of psychiatry, this is not the case, and such a filter is not so easily applied. Psychiatrists hold a unique position in the field of medicine, with more emphasis on subjective individual experience.

The American Psychiatric Association defines Psychiatry as ‘the branch of medicine focused on the diagnosis, treatment and prevention of mental, emotional and behavioural disorders’ [2], and the psychiatrist as ‘a medical doctor who specialises in mental health, qualified to assess both the mental and physical aspects of psychological problems’. This appears at first glance as a role which should fit well into the needs of a modern society. From where then did this subspecialty spawn such staunch opposition, with anti-psychiatry movements questioning both the legitimacy of the specialty, and the validity of their definitions and roles? There is an increased awareness of the gap between diagnostic definitions and the lived personal experience of wellness or illness. The WHO defines health as ‘A state of complete physical, mental and social well-being and not merely the absence of disease or infirmity’ [3]. Is this definition of health the focus of the modern psychiatrist; or is the focus instead on the absence of illness as defined within narrow diagnostic criteria?

## Power within social systems

Michel Foucault (1926–1984) was a French historian and philosopher who had a strong influence in both philosophy and a wide range of humanistic and social science disciplines. His work *Madness and Civilisation: A history of insanity in the age of reason* explored his view of ‘the moral hypocrisy of modern psychiatry’ [4]. He argued that what was presented as ‘an objective, incontrovertible scientific discovery (that madness is mental illness) was in fact the product of eminently questionable social and ethical commitments’ [4]. This may have had some truth in an Irish 1960’s asylum system, which was often used not as a centre for treatment but as a forum for social control, when one in every fifty citizens of the Republic of Ireland was a resident in a psychiatric institution [5]. But is this still the case in a modern, if underfunded, psychiatry system, with individualised care plans and a focus on outpatient treatment in the community? Following the introduction of antipsychotic medications and formalised treatment pathways for previously untreatable diagnoses, Ireland has moved to a system that is no longer reliant on a long-term asylum system.

Foucault was particularly interested in the exercise of power within social systems, and he explored perceived relationships between knowledge and power within medicine in his work *The Birth of the Clinic.* Foucault argued that those in power set the agenda. This is a concept which remains palpable in today’s medical systems in the context of involuntary detention under the Mental Health Act. This power exists only in the initial period of a patients’ illness, and the current system focusses on empowering people once they retain capacity through adequate treatment, a situation only made possible by that initial therapeutic power dynamic. This power does not go unchecked, and the Irish Mental Health Act reflects patients’ rights and adequately allows opportunity for neutral observers to ensure this power is being appropriately used via second opinions and tribunal reviews.

Treatment ideologies and professional approaches within clinical medicine have changed over the years. Nicholas Jewson described the production of medical knowledge as rooted in society, and not within a purely objective science [6]. He described the move from person-orientated to object-orientated to laboratory medicine, which he described in his work *The disappearance of the sick-man from medical cosmology, 1770–1870* [8]. This corresponds with the emergence of Foucault’s described clinical gaze, which no longer views the patient as a whole but instead as a series of disconnected parts.

## Medical systems and socio-cultural contexts

Arthur Kleinman, a psychiatrist and anthropologist, stated that ‘medicine deals with two kinds of reality, “scientific” and “ordinary”; both a biophysical and human science’ [7]. Medical systems do exist in socio-cultural contexts, and ‘the experience of illness is a cultural or symbolic reality’ [7]. Kleinman argued that the narrow traditional medical gaze exists in a system which is ‘an ordered, coherent body of ideas, values, and practices embedded in a given cultural context from which it derives its signification’ [7]. While there is an assumption of power on the behalf of the clinician, ‘the acts of ordering, naming, interpreting, and offering therapy for illness are aspects of symbolic reality common to both the sick individual, the healer, and their society’ [7]. What then is the role of classificatory systems? In psychiatry practice, there is a reliance on the World Health Organisation’s ICD-10 criteria, or the American DSM criteria when applying diagnostic classifications. Having an overarching conceptual understanding of the individuals’ experience helps to guide treatment and provide prognostic clarity that can be comforting and empowering to both patients and their families. This supports the use of diagnostic classification systems despite their flaws and in some cases lack of pure objectivity.

In psychiatry, it is clear that ‘the experience of illness involves feelings, ideas, values, language and non-verbal communication’ [7], all of which are included in a thorough mental state examination. Kleinman argued that there are ‘systematic attempts to restrict medicine’s symbolic reality to a single discipline, psychiatry, peripheral to the central core of medical research interests and practices’ [7]. Kleinmann recognised that psychiatry retains the wider biopsychosocial gaze that is missing in Foucault’s medical gaze, despite its narrow diagnostic classificatory systems.

## Patient narratives of illness

The medical model and its use in the medical gaze ties in with rationalism, black-and-white thinking and beliefs within Western medicine. Byron J. Good, a medical anthropologist, defined the primary role of clinical medicine as ‘the interpretation of the patient’s symptoms by relating them to their functional and structural sources in the body and to underlying disease entities’ [7]. However, he also notes that ‘all medicine joins rational and deeply irrational elements, combining an attention to the material body with a concern for the moral dimensions of sickness and suffering’ [7]. Subjectivity exists for both patient and clinician and inter-rater reliability may not be consistent, and psychiatrists called to provide expert opinion do not always agree in their assessments.

Diagnostic definitions lose their importance if the focus can instead be on individualised treatment of the suffering and distress of each individual, using overarching diagnostic definitions in order to guide these treatments. Good speaks of symptoms as ‘expressions of the experience of distress, communicated as an ordered set of complaints’, but how can this order be ensured? In psychiatry, an understanding of language and culture is integral to definitions of diagnoses themselves. A delusion is defined as ‘a false, unshakeable idea or belief which is out of keeping with the patient’s educational, cultural and social background’ [8]. This is increasingly important in an Irish society which is becoming more diverse.

## Conclusion

Foucault’s concept of the medical gaze is not relevant to the modern psychiatric clinician. The power he speaks of exists only in the initial period of a patients’ illnesses as a treatment tool. While the medical gaze ties clinicians to the use of narrow diagnostic criteria in the treatment of patients, focus should instead be on individualised treatment of the suffering and distress of every individual. It is both increasingly important and difficult for psychiatry services to retain this open perspective to treatment of suffering and distress in a setting where there is increasing pressure secondary to underfunding and increased presentations. Clinicians should continue to focus on helping rather than controlling patients, particularly within a specialty with such direct exposure to the human suffering resulting from trauma, control and societal pressures.

## References

[1] Misselbrook D (2013). Foucault. Br J Gen Pract.

[2] American Psychiatric Association (2021) https://www.psychiatry.org/patients-families/what-is-psychiatry-menu. Accessed 9 May 2021

[3] World Health Organisation (1948) Preamble to the Constitution of the World Health Organization as adopted by the International Health Conference, New York, 19–22 June, 1946. http://whqlibdoc.who.int/hist/official_records/constitution.pdf

[4] Gutting G, Oksala J (2021) Michel Foucault. Stanf Encycl Philos. Summer 2021 Edition.

[5] Kelly B (2019) Hearing voices: the history of psychiatry in Ireland, Irish Academic Press 28657250

[6] Jewson ND (1976). The disappearance of the sick-man from medical cosmology, 1770–1870. Sociology.

[7] Good B, Fischer MMJ, Willen SS et al (2010) A reader in medical anthropology: theoretical trajectories, emergent realities. Wiley-Blackwell

[8] Sims ACP (2003) Symptoms in the Mind: An Introduction to Descriptive Psychopathology. Saunders

